# Conserved mycobacterial sRNA B11 regulates lipooligosaccharide synthesis at posttranscriptional level in *Mycobacterium marinum*

**DOI:** 10.1002/mlf2.70025

**Published:** 2025-08-25

**Authors:** Chuan Wang, Cheng Bei, Yufeng Fan, Qingyun Liu, Yue Ding, Howard E. Takiff, Qian Gao

**Affiliations:** ^1^ Key Laboratory of Medical Molecular Virology (MOE/NHC/CAMS), Shanghai Frontiers Science Center of Pathogenic Microorganisms and Infection, Shanghai Institute of Infectious Disease and Biosecurity, School of Basic Medical Sciences, Shanghai Medical College Fudan University Shanghai China; ^2^ Department of Genetics University of North Carolina at Chapel Hill Chapel Hill North Carolina USA; ^3^ Laboratorio de Genética Molecular, CMBC, Instituto Venezolano de Investigaciones Científicas IVIC Caracas Venezuela

**Keywords:** lipooligosaccharides, MS2‐RNA fishing, mycobacteria, posttranscriptional regulation, sRNA

## Abstract

Extractable glycolipids of mycobacteria, such as lipooligosaccharides (LOSs), play crucial roles in responding to environmental stress and modulating the host immune response. Although the biosynthesis of LOS is likely regulated at multiple levels to ensure proper composition of the cell wall, the key regulators remain unknown. In this study, we investigated B11, a conserved mycobacterial small RNA (sRNA), and found that it post‐transcriptionally regulates LOS synthesis in *Mycobacterium marinum*. Through a combination of RNA‐seq and mass spectrometry screening, we identified specific genes within the LOS synthesis locus that are directly regulated by B11. We confirmed in vivo sRNA‐mRNA interactions using MS2‐tagged RNA affinity purification, and found that B11 utilizes the cytosine‐rich loop of its Rho‐independent transcriptional terminator to interact with guanine tracks adjacent to the ribosome binding sites of its target genes, thereby impeding translation and promoting mRNA degradation. Moreover, deletion of B11 altered the colony morphology associated with LOS composition. These comprehensive functional studies of the mycobacterial sRNA B11 reveal sRNA‐based regulation of LOS synthesis, providing new insights into the regulatory mechanisms controlling the biosynthesis of the complex mycobacterial cell wall.

## Impact statement

Despite the identification of mycobacterial sRNAs over a decade ago, their functions and regulatory mechanisms have remained largely unexplored. This study presents a comprehensive analysis of mycobacterial sRNAs, utilizing robust experimental approaches to identify key target genes. Our findings demonstrate how the conserved sRNA B11 regulates the synthesis of lipooligosaccharides (LOSs), crucial components for environmental stress response and host immune modulation. This study highlights the evolving functional roles of sRNAs across different mycobacterial species, offering valuable insights into their diverse regulatory functions.

## INTRODUCTION

Mycobacteria possess a cell envelope that plays an important role in host–pathogen interactions and susceptibility to antibiotics^1^. The defining feature of the mycobacterial cell envelope is a complex cell wall outside of the typical bacterial plasma membrane, termed the mycomembrane, which is composed of noncovalently linked peptidoglycan, arabinogalactans, and mycolic acids^2^. The outer leaflet of the mycomembrane contains the extractable glycolipids, including a variety of elements as glyco‐peptidolipids (GPLs), phenolic glycolipids (PGLs), trehalose mycolate (cord factor), and lipooligosaccharides (LOSs). Similar to the diverse O polysaccharides found in the lipopolysaccharides (LPS) of Gram‐negative bacteria, the structures of mycobacterial glycolipids are species‐specific. For example, more than 30 LOSs have been identified in different mycobacterial species, including *M. kansasii*, *M. canettii,* and *M. marinum* ^3^, and all have a trehalose core attached to the varied lipidic moieties^4^. The diverse mycobacterial glycolipids confer phenotypic differences and are also implicated in the pathogenicity of some mycobacteria. For example, the smooth–rough (S/R) colony morphotype variation is attributed to the presence or absence of GPL in *M. abscessus* and LOS in *M. marinum*, *M. canettii,* and *M. smegmatis* ^5^, ^6^. The biosynthesis pathways of these glycolipids are well‐defined, and their synthesis has been shown to be controlled at the transcriptional level by regulatory factors such as Lsr2 and serine‐threonine kinases^7^, ^8^, but the exact mechanisms by which this regulation responds to internal and external cues during growth and stasis remain unknown.

Posttranscriptional control by small regulatory RNAs (sRNAs) is a key element in the regulation of many biological processes, including cell wall synthesis^9^. The majority of the currently known sRNAs are 50–250 nucleotides in length and typically regulate their target mRNAs through imperfect base pairing near the ribosome binding sites (RBSs), thereby both inhibiting translation initiation and promoting mRNA degradation^10^, ^11^, ^12^. Alternatively, base‐pairing outside of the RBSs can promote translation by altering the mRNA's secondary structure to facilitate ribosome binding, or by masking RNase recognition motifs to reduce mRNA degradation^13^, ^14^. In addition to regulation at the posttranscriptional level, sRNA base‐pairing can also modulate transcription by suppressing premature Rho‐dependent transcription termination^15^.

Despite sharing similar base‐pairing mechanisms, sRNA‐mediated regulation varies among different bacterial species. In Gram‐negative bacteria, including *Escherichia coli* and *Salmonella*, regulation of most sRNAs is dependent on RNA chaperons Hfq or ProQ, which can accelerate sRNA‐mRNA annealing and protect sRNAs from RNase degradation^16^, ^17^. In Gram‐positive bacteria, however, the role of Hfq and ProQ homologs in sRNA‐mediated regulation is unclear. In low GC‐content Gram‐positive bacteria, such as *Bacillus subtilis* and *Staphylococcus aureus*, the deletion of Hfq had no impact on sRNA‐mediated regulation^18^, and the genomes of high GC‐content bacteria, such as mycobacteria, lack Hfq or ProQ homologs. It is possible that the strength of base‐pairing interactions with GC pairs makes the RNA chaperons unnecessary for sRNA‐mRNA annealing, or alternatively, the high GC‐content bacteria may have distinct RNA chaperones that have yet to be identified. Although several functional sRNAs have been described in different mycobacterial species, including *M. tuberculosis* ^19^, ^20^, ^21^, ^22^, ^23^, ^24^, ^25^, studies to define their biological function and regulatory mechanisms are scarce.

In this study, we explored the biological function and regulatory mechanisms of sRNA B11 in *M. marinum*. By using different methods to discover B11‐targets, we found that B11 controls several key genes in the LOS biosynthesis locus, which is distinct from the regulatory roles reported for B11 orthologues in *M. smegmatis* and *M. abscessus* ^26^, ^27^. Furthermore, we found that *M. marinum* strains lacking B11 display a clear colony phenotype, suggesting that B11 acts as a negative posttranscriptional regulator of LOS synthesis. The work presents a comprehensive functional study of mycobacterial sRNA, highlighting its role in RNA‐based gene regulation involved in the synthesis of mycobacterial cell wall components.

## RESULTS

### B11 is an abundant and stable sRNA in *M. marinum*

**Figure 1:**
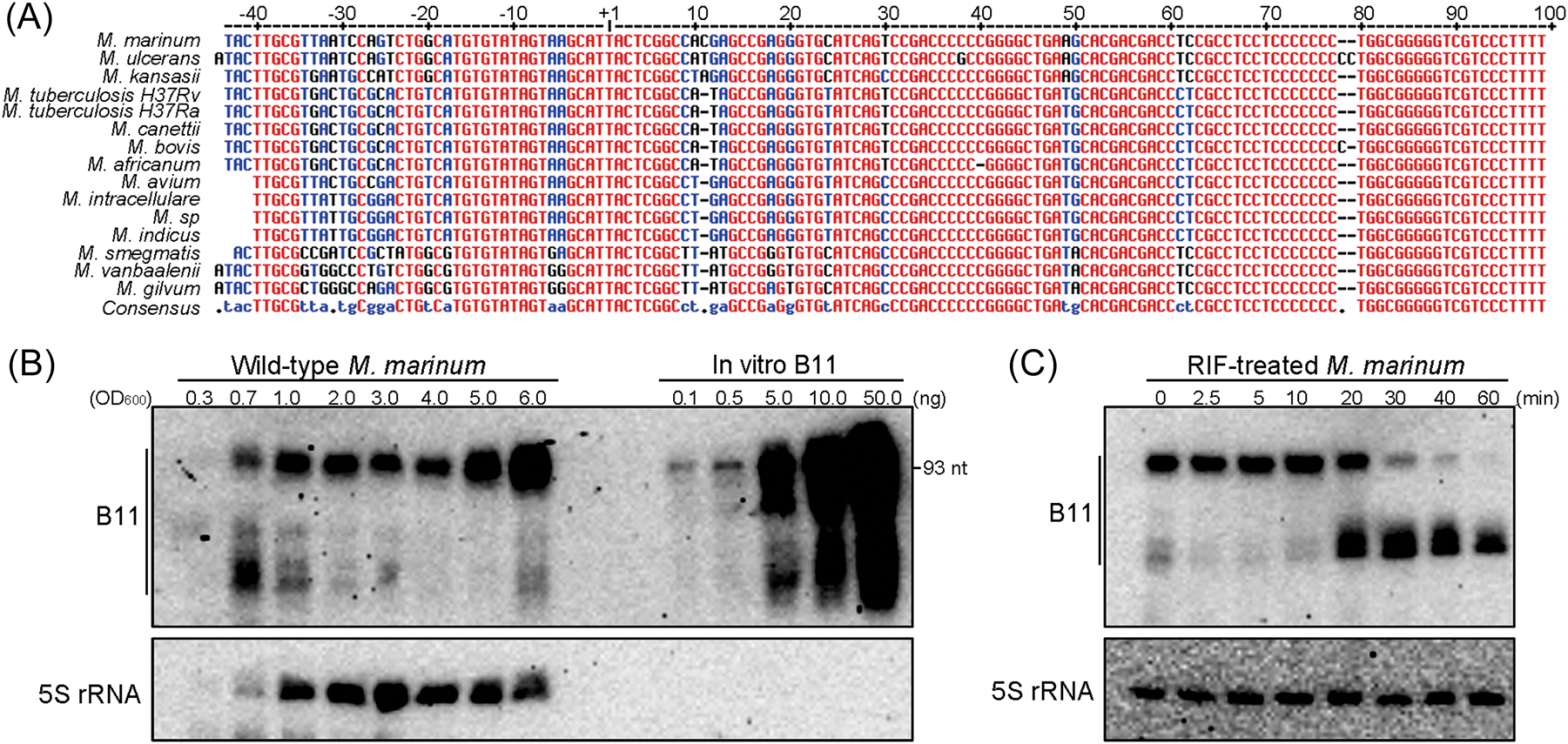
B11 is a conserved, abundant and stable sRNA in *Mycobacterium marinum*. (A) Nonredundant alignment of the *b11* genes, including the upstream promoter regions, in different mycobacteria. All nucleotides are colored according to their degree of conservation: red, high conservation; blue, partial conservation; black, little or no conservation. “+1” marks the transcriptional start site. (B) Expression levels of B11 in an *M. marinum* wild‐type strain through standard 7H9‐OADC growth. Northern blot analysis of total RNA isolated from wild‐type *M. marinum* grown to the indicated OD_600_ was performed. In vitro transcribed B11 was loaded as a standard to calculate the amount of in vivo B11. 5S rRNA was used as loading controls. (C) Stability of B11. Wild‐type *M. marinum* strain was grown to OD_600_ ~ 1.0 and treated with rifampicin (RIF) at a final concentration of 200 μg/ml. Samples were collected at indicated time points and analyzed by northern blot.

B11 is a 93‐nt sRNA that was first identified in the *Rv3660‐Rv3661* intergenic region of *M. tuberculosis* ^19^. The nucleotide sequence of B11 is more than 90% conserved in mycobacteria (Figure 1A), including a −10 motif (TATAGT) that matches the consensus sequences of sigma factor A, promoter typically found upstream of housekeeping genes. Because studies in *M. tuberculosis* require strict biosafety infrastructure, we chose to work with the more tractable *Mycobacterium marinum* as a laboratory model to explore the biological function of B11. Transcriptomic analyses in different mycobacteria have shown that B11 is one of the most abundant sRNAs^21^, ^22^, ^28^. To confirm whether this was also true in *M. marinum*, we evaluated the expression of B11 during different growth phases in 7H9 rich medium using northern blot. We found that B11 expression was low during the lag phase at OD_600_ ~ 0.3, but increased during the early exponential phase (OD_600_ ~ 0.7) and remained high for at least 5 days while the cultures had reached an OD_600_ of 6.0 (Figure 1B). Intriguingly, we found that 5S RNA, whose presence is often used as a reference in northern blot of many bacteria, exhibited an expression pattern similar to B11, with low expression at lag phase and then steady levels beginning with exponential phase growth. This pattern may result from the low processing rate of mycobacterial ribosomal RNA precursor transcript during early growth^29^. To achieve a more accurate quantification of B11 levels during early growth, we performed qRT‐PCR and found a similar expression pattern compared to the northern blot data. Specifically, B11 levels were induced by ~10 fold starting at OD_600_ ~ 1.0, compared to the RNA levels at OD_600_ ~ 0.3 and OD_600_ ~ 0.7 (Figure S1). Moreover, quantification with in vitro‐generated B11 RNA suggested that B11 accounts for ~0.1% of total RNA by weight (~5 ng of 5000 ng) at stationary phase (OD_600_ ~ 6.0), corresponding to approximately 150‐600 copies/cell. When compared with sRNAs such as SdsR in *E. coli* and *Salmonella*, which are present in ~300 copies/cell, or RaiZ present in ~50 copies/cell^17^, ^30^, B11 appears to be relatively abundant in *M. marinum*. We also determined the stability of B11 by arresting transcription initiation with rifampicin and found that the half‐life of B11 was more than 20 min (Figure 1C). When compared to the average half‐life of 9.5 min for *M. tuberculosis* mRNA and 5.2 min for *M. smegmatis* mRNA^31^, B11 appeared to be quite stable. We found that degradation of B11 resulted in a processed isoform that was resistant to further decay, which was observed during standard growth (Figure 1B). The high abundance and prolonged stability of B11 suggested that it could play an important role in the biology of mycobacteria.

### Deletion of B11 alters *M. marinum* colony morphology

**Figure 2:**
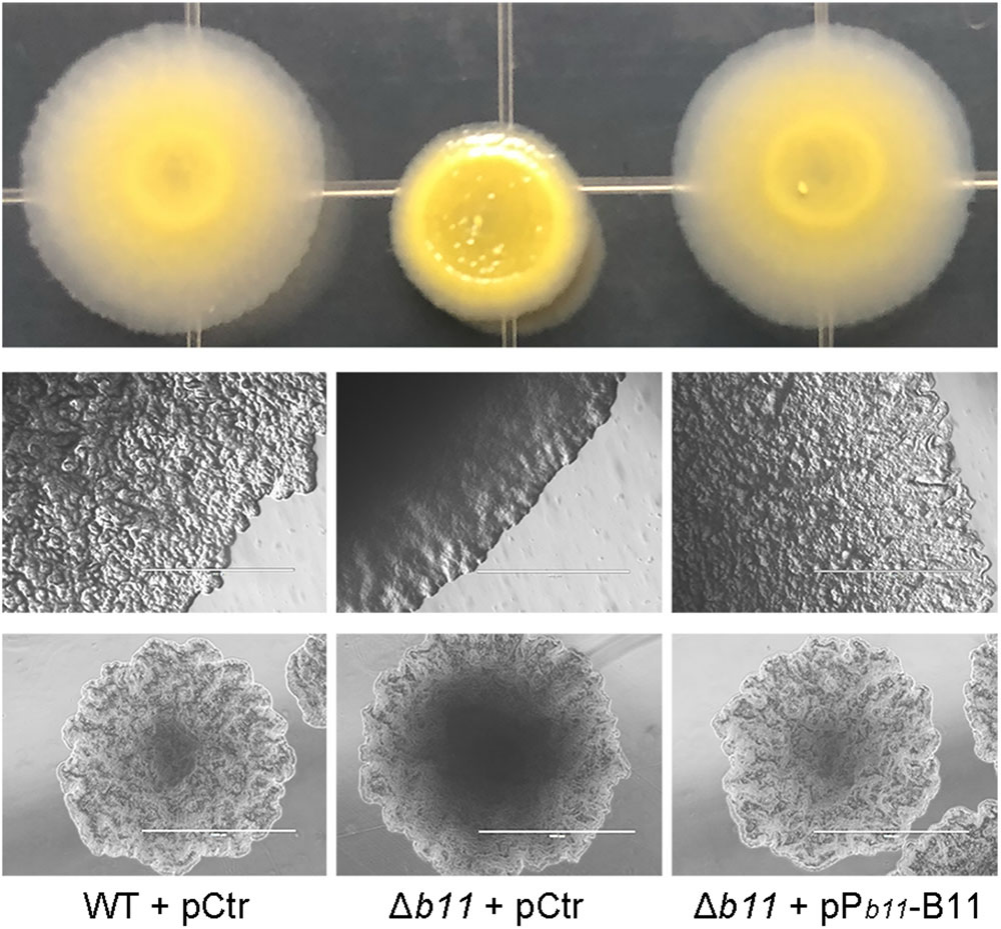
Deletion of B11 alters colony morphology of *M. marinum*. Colony morphology of the indicated *M. marinum* strains in 7H10 agar is shown. The strains were grown to OD_600_ ~ 1.0 in 7H9‐OADC, serially diluted and plated on 7H10‐OADC agar, incubated for 5 days, and visualized by camera (upper) or microscopy (middle). Single colonies of the indicated strains were also checked by microscopy (lower). The wild‐type strain, WT + pCtr; B11‐deleted strain, Δ*b11* + pCtr; and B11 complemented strain, Δ*b11* + pP_*b11*_‐B11. Scale bar, 1000 μm.

To identify the function of B11 in *M. marinum*, we constructed a B11‐deleted strain (Δ*b11*) by replacing nucleotides 36–91 of B11 in the *M. marinum* genome with a kanamycin‐resistance cassette, using a modified allelic exchange method based on temperature sensitive plasmid pPR27^32^. The deletion was confirmed by northern blot showing no expression of B11 in the deleted strain (Δ*b11*) during standard bacterial growth (Figure S2A). A slight and nonsignificant growth difference was observed for the Δ*b11* strain carrying empty control plasmid (Δ*b11* + pCtr) during exponential phase growth in 7H9‐OADC media, which was reversed by complementation of B11 expressed from its native promoter in multi‐copy plasmid pSMT3 (Δ*b11* + pP_*b11*_‐B11) (Figure S2B). Intriguingly, the Δ*b11* strain displayed a significantly altered colony morphology on 7H10‐OADC agar plates, with a reduction in the broad translucent border halo characteristic of wild‐type *M. marinum* colonies (Figure 2). Complementation of Δ*b11* with pP_*b11*_‐B11 plasmid fully restored the wild‐type colony morphology. As changes in mycobacterial colony morphology usually reflect alterations in the composition of cell wall glycolipids, the altered morphology of the Δ*b11* strain suggests that B11 is involved in cell wall synthesis in *M. marinum*.

### B11 represses the expression of genes from the LOS biosynthetic locus

**Figure 3:**
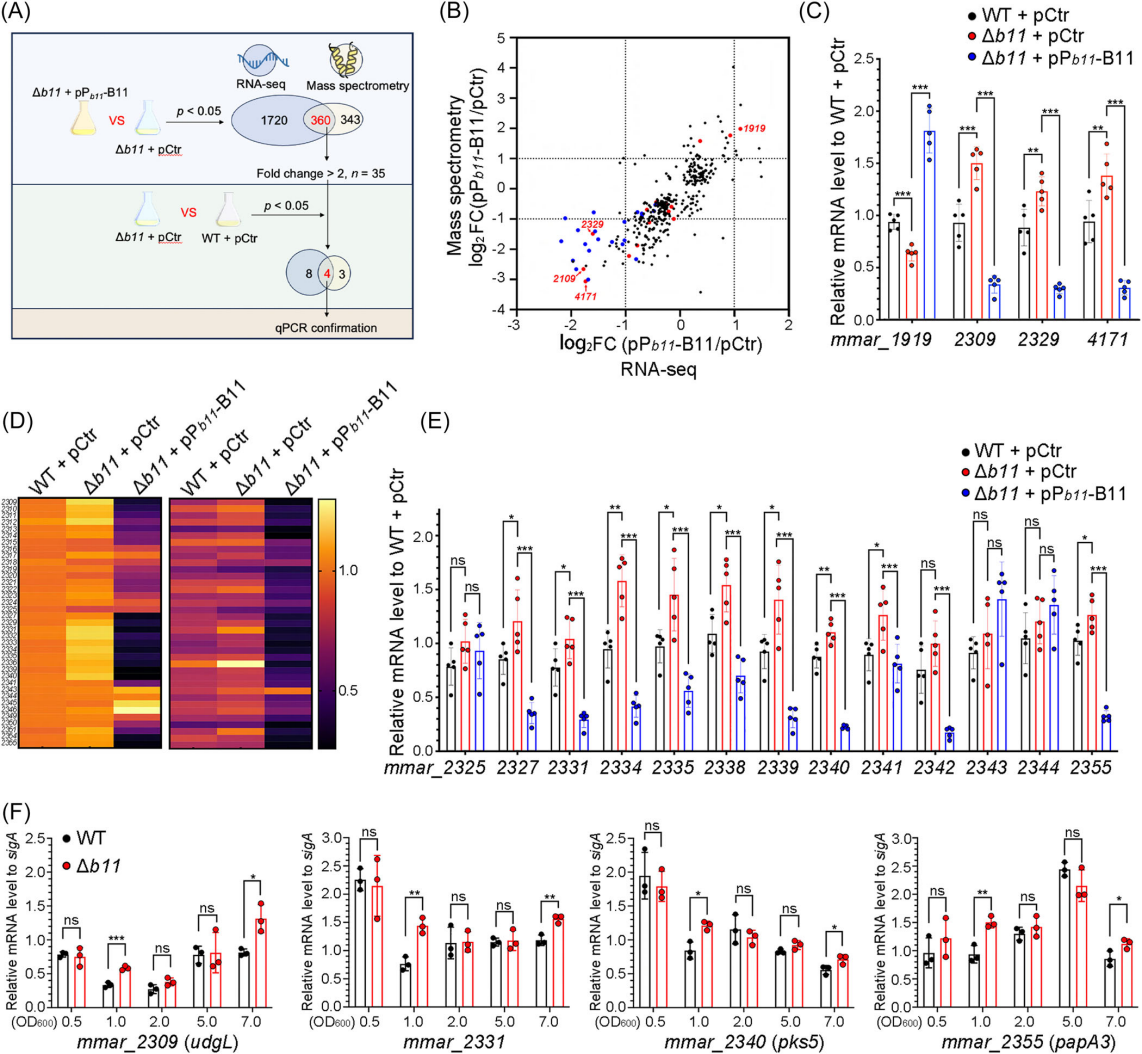
B11 represses the expression of genes from lipooligosaccharide (LOS) biosynthetic locus. (A) Schematic overview of the workflow for transcriptomic and proteomic analysis. Total RNA and protein from the indicated strains grown to OD_600_ ~ 1.0 were analyzed by RNA sequencing and mass spectrometry. (B) Scatter plot displaying the log_2_(fold change) for genes with significant changes (*p* < 0.05) in both the RNA sequencing (*x*‐axis, *n* = 2) and mass spectrometry data (*y*‐axis, *n* = 3) between B11‐deleted (Δ*b11* + pCtr) and complemented (Δ*b11* + pP_*b11*_‐B11) strains. 16 genes displaying significant gene expression changes (*p* < 0.05) between wild‐type and B11‐deleted strains in both RNA sequencing and mass spectrometry are marked in red. Genes from the LOS biosynthetic locus (*mmar_2309–2355*) are marked in blue. Dashed lines refer to twofold changes. (C) Expression of four screened genes in the indicated strains confirmed by qRT‐PCR. *sigA* was set as the reference gene for data analysis, and expression levels were normalized to one sample from the WT + pCtr group. Error bars indicate standard deviations (*n* = 5). (D) Heatmap displaying relative RNA and protein levels of genes from the *M. marinum* LOS locus (*mmar_2309–2355*), detected by RNA sequencing (left) and mass spectrometry (right). Expression levels were normalized to one sample from the WT + pCtr strain. Genes with undetected values were filtered. (E) Expression of selected genes from the LOS locus in the indicated strains measured by qRT‐PCR and normalized to one sample from the WT + pCtr group. Error bars indicate standard deviations (*n* = 5). (F) Regulation of selected genes by B11 during different growth phases (*n* = 3). *p < 0.05, **p < 0.01, ***p < 0.001 and ns, no significant difference in a two‐tailed *t* test.

To explore the molecular mechanism by which B11 regulates cell wall composition, we performed RNA sequencing and mass spectrometry to analyze differences in the transcriptomes and proteomes between the wild‐type (WT + pCtr), B11‐deleted (Δ*b11* + pCtr), and B11 complemented (Δ*b11* + pP_*b11*_‐B11) strains. RNA and protein samples were collected during early exponential phase (OD_600_ ~ 1.0), when the level of B11 expression in wild‐type strains is stable. To identify B11‐regulated genes, we performed two rounds of screening with different datasets (Figure 3A). The first‐round focused on identifying genes whose expression differed between B11‐deleted and complemented strains with overexpressed B11. As bacterial sRNAs typically block mRNA translation initiation by interacting with the RBS, mRNAs targeted by sRNAs are usually more susceptible to RNase degradation due to the loss of ribosome protection. Consequently, direct targets of sRNAs often exhibit reduced levels at both the RNA and protein levels upon sRNA overexpression. Therefore, we focused on the overlap between RNA‐seq and proteomics data to identify genes whose expression changes may directly result from B11 regulation.

We identified 360 genes that surpassed the difference threshold (*p* < 0.05) in both the RNA sequencing and mass spectrometry datasets (Figure 3B, Table S1), and the degree of the differences in the mRNA and protein levels were significantly correlated (*p* < 0.0001, *r* ^2^ = 0.3378, Simple linear regression). Among these 360 genes, 35 exhibited greater than twofold differences in both RNA and protein levels. A second round of selection focused on comparing the mRNA and protein levels of these 35 genes in the wild‐type versus B11‐deleted strains, to identify genes regulated by B11 at physiological expression levels. This analysis identified four candidates with significant differences by more than twofolds (*p* < 0.05): *mmar_1919*, *mmar_2309*, *mmar_2329*, and *mmar_4171*. Lastly, qRT‐PCR on RNA samples prepared from five additional biological replicates confirmed the expression differences of these four candidate genes (Figure 3C).

We noticed that two of the four screened candidates, *mmar_2309* and *mmar_2329*, are located in the *mmar_2309‐ mmar_2346* genomic locus that spans approximately 60 kb (Figure S3) and is found only in *M. marinum*. This locus contains 38 genes encoding enzymes involved in the synthesis of LOS, which have been associated with the colony morphology of other mycobacteria species^33^. Of note, both RNA sequencing and mass spectrometry revealed that nearly half of the genes in this locus were downregulated when B11 was overexpressed (Figure 3D, Table S2), as were other genes close to this locus, *mmar_2350–2355*, which are also involved with LOS biosynthesis. Nine of the LOS‐associated genes had higher mRNA levels in the Δ*b11* strain compared with wild‐type strains, as shown by RNA sequencing and qRT‐PCR on additional biological replicates (Figure 3D,E). Moreover, the differences in the expression of these genes between the Δ*b11* and wild‐type strains were seen in the early exponential (OD_600_ ~ 1.0) and stationary phases (OD_600_ ~ 7.0), but no differences were observed during mid (OD_600_ ~ 2.5) and late exponential phases (OD_600_ ~ 5.0), indicating that B11 regulation is growth phase dependent (Figure 3F). Taken together, these results suggest that B11 regulates the biosynthesis of LOS by targeting multiple genes in an *M. marinum*‐specific genomic locus.

### Identification of B11‐targets by in vivo MS2 affinity purification

**Figure 4:**
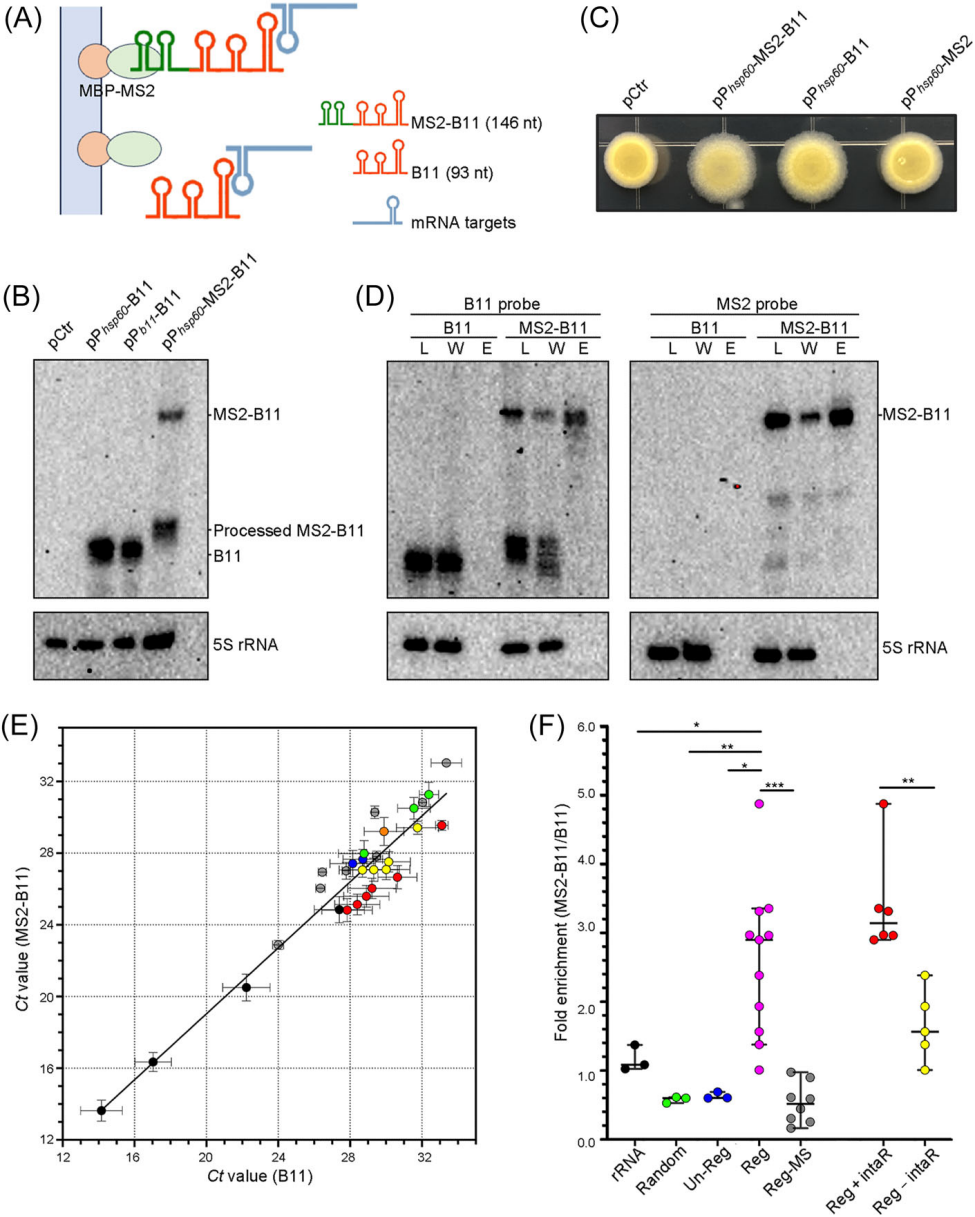
Identification of B11‐targtes by in vivo MS2 affinity purification. (A) Schematic illustrating affinity purification of MS2 aptamer‐tagged B11 to identify mRNA targets. The MS2 RNA sequence was fused to the 5′ end of B11 and expressed in vivo from a plasmid in a B11‐deleted strain of *M. marinum*. MS2‐B11 RNA retains its capability for mRNA binding and can be captured by the MBP‐MS2 protein complex. (B) Northern blot analysis of total RNA isolated from the B11‐deleted strain carrying the indicated plasmids. 5S RNA was used as loading controls. (C) Morphology of B11‐deleted strain carrying the indicated plasmids in 7H10 agar. (D) Northern blot analysis of RNA isolated from crude lysate (L), lysate washed through MBP column (W), and eluted samples (E) from B11‐deleted strain carrying either P*_hsp60_*‐MS2‐B11 or P*_b11_*‐B11 plasmids, and probed for B11 (left) or MS2 (right). 5S RNA was used as loading controls. (E) Correlation of qRT‐PCR *C*t values of selected genes in RNA from elution samples of B11 deleted strains of *M. marinum* that contain plasmids with either B11 or MS2‐B11. The dots indicate: genes that are regulated by B11 either with (red dots, Reg + IntaR, *n* = 6) or without (yellow dots, Reg – IntaR, *n* = 5) IntaRNA predicted binding regions; genes from the LOS biosynthesis locus that are not regulated by B11 (blue dots, un‐Reg, *n* = 3); randomly chosen genes from previous work in our laboratory (green dots, random, *n* = 3); regulated proteins screened by mass spectrometry (gray dots, Reg‐MS, *n* = 8); and rRNAs (black dots, *n* = 3). Simple linear regression analysis was performed with *C*t values of all tested genes, and the regression line is shown. (F) Fold enrichment after affinity purification for genes in the different groups, calculated after normalization. The dots from the indicated groups are marked with the same colors as in panel E, with the addition of B11‐regulated LOS synthesis genes (collection of group Reg + IntaR and Reg − IntaR), which are represented by magenta dots (Reg, *n* = 11). Each group is represented by a marked bar indicating the median value along with a 95% confidence interval. *p < 0.05, **p < 0.01, ***p < 0.001, in a two‐tailed *t* test.

Bacterial sRNAs commonly mediate posttranscriptional regulation through base pairing with the target mRNA^34^, ^35^. The presence of multiple B11‐regulated genes in the LOS biosynthetic locus suggests that B11 may not regulate each of these genes individually, but may target only genes encoding enzymes upstream in the LOS biosynthesis pathway. To exclude this possibility and confirm the genes that B11 regulates directly through sRNA‐mRNA binding, we used MS2 affinity purification to capture sRNA‐mRNA interactions in vivo^36^, ^37^. In this approach, an MS2 RNA aptamer was fused to the 5′ end of B11, expressed from the strong *hsp60* promoter on the multi‐copy plasmid pSMT3. Since the MS2 region of the MS2‐B11 RNA can bind to the MS2 coat protein, mRNAs that interact with MS2‐B11 RNA will be affinity purified from total RNA after the flow‐through by forming the mRNA‐sRNA‐MS2 protein complex (Figure 4A). MS2‐B11 was successfully expressed as a 146 nt fragment (51 nt MS2, 2 nt UU linker, and 93 nt B11), accompanied by additional processed fragments of sizes similar to untagged B11 (93 nt) (Figure 4B). The MS2‐B11 RNA complemented the Δ*b11* strain, restoring the wild‐type colony morphology, demonstrating that the B11 sequence remained functional (Figure 4C). Northern blot showed that the quantity of the MS2‐B11 construct was reduced in the affinity flow‐through and recovered in the elution sample (Figure 4D), indicating successful capture by affinity purification. In contrast, the level of untagged B11 remained similar in the original lysate and the affinity flow‐through and was undetected in the elution sample.

To confirm target enrichment with the MS2‐B11 affinity purification, we selected 11 of the qRT‐PCR validated gene targets in the LOS biosynthetic locus (Figure 3C,E) to check their mRNA abundance in the elution samples of cells expressing MS2‐tagged or untagged B11. In addition, we selected the 10 genes encoding abundant proteins that mass spectrometry showed to have the greatest difference in protein levels when B11 was overexpressed (Figure S4), to evaluate which method was more effective to capture the direct binding targets. Only 2 of these 10 (*mmar_2309* and *mmar_4171*) showed similar B11 regulation in the RNA sequencing data. To minimize bias from systematic experimental variation, we used three sets of reference genes to normalize the qRT‐PCR results: three genes in the LOS biosynthesis locus that are not regulated by B11 (*mmar_2325*, *mmar_2343*, and *mmar_2344*); three genes randomly chosen from previous unpublished work in our laboratory (*mmar_3700*, *mmar_1863*, and *mmar_4219*); and four genes commonly used as references for qRT‐PCR (three rRNAs and *sigA*). Simple linear regression analysis with the *Ct* values of all 29 tested genes showed a similar distribution in the MS2‐B11 and B11 samples (slope = 0.9202 ± 0.025 and *r* ^2^ = 0.9003, Figure 4E). We then used quantile normalization to replace each *Ct* value with the average of that quantile across all tested genes^38^. The degree of enrichment obtained by affinity purification was calculated by comparing the normalized *Ct* values in the eluted samples from the Δ*b11* strains expressing either untagged B11 or tagged MS2‐B11. The fold changes (MS2‐B11/B11) for the normalized *Ct* median values were 1.085 (median value) for rRNAs, 0.602 for the randomly chosen genes, 0.607 for non‐B11 regulated genes, 0.520 for mass spectrometry screened genes, and 2.898 for the B11‐regulated LOS synthesis genes, which were significantly enriched (*p* < 0.05) when compared to any of the other gene groups (Figure 4F).

We then used IntaRNA to predict the putative B11 binding sites in the 11 screened targets from the LOS synthesis locus. The five genes (*mmar_2330*, *mmar_2334*, *mmar_2335*, *mmar_2338*, and *mmar_2341*) predicted to lack B11 binding sites had a median enrichment ratio of 1.565, whereas the six genes predicted to contain B11 binding sites (*mmar_2309*, *mmar_2331*, *mmar_2355*, *mmar_2340*–*2339 operon* and *mmar_4171*, Figure S5) had a significantly higher median enrichment ratio of 3.139 (Figure 4F). This difference in fold enrichment suggests that B11 regulates the expression of these six targets directly by classical sRNA base‐pairing in vivo. Additionally, the median enrichment for the abundant proteins, identified by mass spectrometry as having the greatest differences upon B11 overexpression, was only 0.520, and none of the genes were predicted to have B11 binding sites, indicating that they are not directly regulated by B11.

To further confirm that B11 regulates the screened targets through base‐pairing, we performed electrophoretic mobility shift assays (EMSA) to assess whether B11 can bind to these targets in vitro. We observed visible shifts for the 5′ UTRs of mRNA from five transcripts with predicted B11‐binding sites (*mmar_2309*, *mmar_2331*, *mmar_2355*, *mmar_4171*, and the *mmar_2340–2339 operon*, Figure S6A), but no clear shift for the B11‐repressed genes lacking predicted binding sites (*mmar_2334*, *mmar_2335*, *mmar_2338*, and *mmar_2341*). Upon examining the binding affinity of B11 for these targets (Figure S6B–F), we found that the B11‐mRNA interaction affinities were in a range consistent with previously reported B11‐mRNA interactions in *M. smegmatis* ^26^, although they were lower compared to the typical sRNA‐mRNA interactions observed in *E. coli* and *Salmonella* ^17^. Among the targets, *mmar_2309* exhibited the highest affinity (Figure S6B), with a saturated shift observed at a B11: mRNA ratio of 50:1. In contrast, the shifts for the other four targets were saturated at a B11: mRNA ratio of approximately 150:1. Interestingly, although the B11‐binding sites on the 5′ UTRs of *mmar_2309* and *mmar_2331* share the same sequence, they displayed distinct binding affinities. This suggests that other factors, such as RNA secondary structures surrounding the binding regions, may also influence the binding of B11.

### Cytosine‐rich loop in the transcriptional terminator of B11 is crucial for regulation

**Figure 5:**
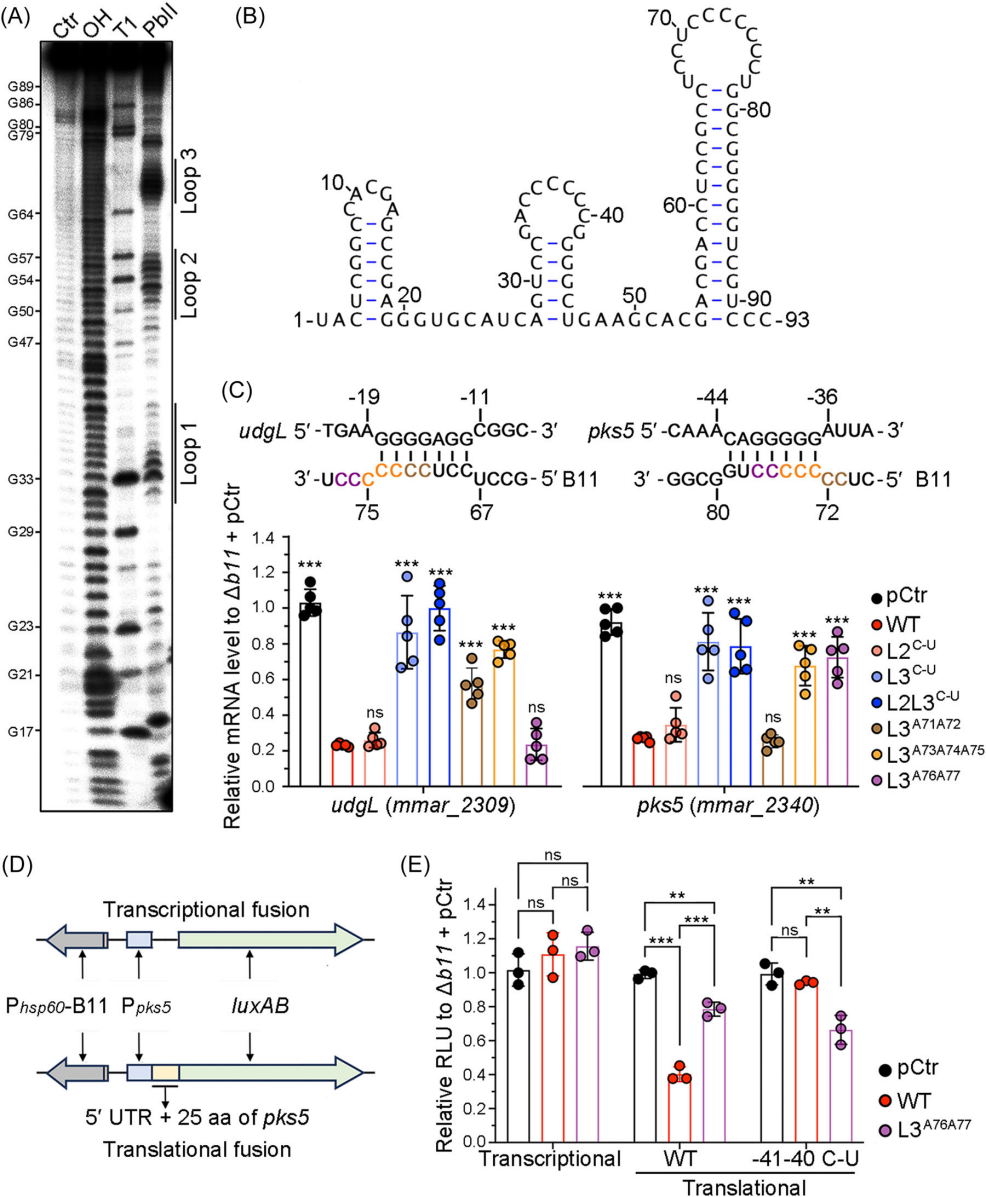
B11 regulates its targets through base pairing. (A) In vitro structure probing of B11 RNA using 5′ end‐labeled B11 RNA with lead (II) acetate (PbII), RNase T1 (T1), and alkaline ladders (OH) to map cleaved fragments. Positions of G‐residues are indicated. (B) Illustrated secondary structure of B11 based on structure probing data. (C) Expression of selected B11 targets *udgL* (*mmar_2309*) and *pks5* (*mmar_2340*) in the B11‐deleted strain carrying different plasmids, confirmed by qRT‐PCR. *sigA* was used as the reference gene for data analysis, and expression levels were normalized to one sample from the Δ*b11* + pCtr group. Error bars indicate standard deviations (*n* = 5). ***p < 0.001, and ns refers to no significant difference between the indicated group and the Δ*b11* + pB11‐WT group, two‐tailed *t*‐test. Predicted RNA duplex formations between B11 and *udgL*/*pks5* mRNA 5′ UTR are shown above. Distances from the +1 site of B11, or the start codons of mRNA sequences, are indicated. Nucleotides used for mutation analysis are marked in colors. (D) Schematic showing the transcriptional and translational reporter system used for the bioluminescence assay. (E) Relative luminescence unit (RLU) of the B11‐deleted strain carrying the indicated transcriptional and translational reporter plasmids. The results were normalized to one sample from the Δ*b11* + pCtr group. Error bars indicate standard deviations (*n* = 3). **p < 0.01, ***p < 0.001, and ns refers to no significant difference, two‐tailed *t*‐test.

To explore the mechanistic details of B11‐mediated repression, and confirm the region for base‐pairing, the secondary structure of B11 was determined using in vitro structure‐probing with single‐strand‐specific Pb (II). This analysis showed that B11 is highly structured with 3 intra stem‐loops (Figure 5A,B), two of which are cytosine‐rich: six cytosine residues in loop‐2 and nine in loop‐3. In addition, loop‐3 has the structure of a typical Rho‐independent transcriptional terminator, with a GC‐rich hairpin followed by a uridine‐rich segment^39^. IntaRNA predicted that all 5 confirmed B11 targeted transcripts harbor guanine‐tracks near their RBS, presumably allowing interaction with B11 cytosine‐rich loop‐2 or ‐3 (Figures 5C and S4). To identify which loop is involved in B11‐mediated regulation, we replaced the cytosine residues in loop‐2 and ‐3 with uridines. Replacing the cytosines in loop‐2 did not reduce B11 expression, whereas replacing the cytosines in loop‐3 significantly decreased the levels of B11 (Figure S7A). Moreover, replacing the cytosine residues in loop‐2 with uridines had no impact on the complementation of cell morphology (Figure S7B) or the regulation of selected target genes *udgL* (*mmar_2309*) and *pks5* (*mmar_2340*) compared to wild‐type B11 (Figure 5C). In contrast, replacing the cytosines in loop‐3 abolished B11‐mediated repression of target genes and failed to restore the morphological changes. When both loop‐2 and ‐3 were mutated, the effects were identical to those observed when only loop‐3 was mutated, suggesting that loop‐3 is the critical region for B11‐mediated regulation.

The reduced B11 levels in the loop‐3 mutation may also account for the loss of B11‐mediated regulation. To distinguish whether the observed loss of regulation was due to reduced B11 levels or the mutated nucleotides, we sequentially mutated the cytosines in loop‐3 and assessed their effects on B11 expression as well as the regulation of representative targets. Mutating cytosines to guanine could result in intra‐loop G‐C base pairing, while mutating a limited number of cytosines to uridine might preserve B11‐mRNA interaction by forming non‐Watson‐Crick G‐U base‐pairing. To avoid these complications, we mutated the cytosines to adenosines. We still observed reductions in B11 levels in the three tested mutant strains (C71C72‐A71A72, C73C74C75‐A73A74A75, and C76C77‐A76A77; Figure S7A); however, the levels were significantly higher than when the entire loop‐3 was mutated. In addition, the B11 levels within these three mutant strains were consistent. Since IntaRNA predictions suggest that *udgL* and *pks5* pair with different regions of loop‐3 (Figure 5C), we were able to compare the target mRNA levels in these mutant strains, which had similar B11 levels but different mutated regions. We found that mutating the last two cytosines (C76C77‐A76A77) did not significantly affect B11‐mediated regulation of *udgL* (Figure 5C), as these two cytosines are not predicted to be involved in interactions with this target. By contrast, mutations in C71C72‐A71A72 and C73C74C75‐A73A74A75 significantly reduced B11‐mediated regulation, suggesting that these cytosines are important for regulation. For *pks5*, mutations in the first two cytosines (C71C72‐A71A72) had no effect on B11‐mediated regulation, while mutations in the other cytosines (C73C74C75‐A73A74A75 and C76C77‐A76A77) reduced the regulation, consistent with the predicted base‐pairing sites. Taken together, these data support the conclusion that the C‐rich loop‐3 is responsible for B11‐mediated interaction and regulation of its targets.

To further confirm that B11‐mediated repression occurs at the posttranscriptional level, we created a translational reporter system (Figure 5D). In this reporter plasmid, B11 was expressed from the strong constitutive *hsp60* promoter on plasmid pSMT3. The native promoters of the gene target *pks5*, along with its 5′ UTRs and the first 25 amino acids, were fused to the second amino acid of *luxAB* to yield a translational fusion. B11 and the *luxAB* fusion were expressed from different strands of the plasmid to exclude potential *cis*‐acting regulation. Additionally, the native promoter of *pks5* alone was also fused to *luxAB* with its own RBS, creating a transcriptional reporter system where *luxAB* expression is solely dependent on the target gene promoter. We chose *pks5* for this analysis as the B11‐binding site in *pks5* is upstream of its ribosomal binding site, which allowed us to create compensatory mutations in the mRNA without affecting translation.

Bioluminescence assays showed that B11 had no significant effect on the transcriptional reporter system P*pks5*‐*luxAB*, excluding any regulation in the transcriptional level (Figure 5E). In contrast, B11 significantly reduced the relative luminescence units (RLUs) of the *pks5* 5′ UTR::*luxAB* translational fusion by 2.48 ± 0.22 fold, compared to the Δ*b11* strain carrying the control plasmid without B11. Mutation of C76C77‐A76A77 in B11 loop‐3 reduced this regulation to 1.27 ± 0.10‐fold, indicating that the regulation is base‐pairing dependent. Conversely, compensatory mutations in the *pks5* mRNA blocked regulation by wild‐type B11. Combining the mutated B11 C76C77‐A76A77 variant with the respective mutated *pks5* (G‐41G‐40 to U‐41U‐40) restored *luxAB* repression, although to a reduced extent (1.51 ± 0.25‐fold) compared to the wild‐type B11–*pks5* interaction, likely due to the A‐U base pairing in the compensatory mutation being less stable than the original G‐C base pairing. Taken together, these data strongly suggest that B11 regulates its targets at the posttranscriptional level, through the seed region located in the C‐rich track of the loop‐3.

### RNase E is likely involved in B11‐mediated mRNA degradation

In Gram‐negative bacteria such as *E. coli* and *Salmonella*, base pairing around the mRNA RBS reduces ribosome binding frequency and leads to mRNA degradation, mainly by endoribonuclease RNase E^12^. To determine whether B11 binding in *M. marinum* similarly leads to degradation of target mRNAs, we evaluated B11‐mediated mRNA degradation in a strain where *rne* (*mmar_3768*, the RNase E coding gene) transcription was interfered with by an anhydrotetracycline (ATc)‐induced CRISPRi knockdown system^40^. At 24 h after ATc induction, *rne* mRNA levels were reduced by approximately 10‐fold (Figure S8). Correspondingly, the fold changes in the mRNA levels of the B11 target genes (*udgL*, *pks5*, *mmar_2331*, and *papA3*) between cells carrying the B11 overexpression plasmid (pB11) and the empty control (pCtr) were decreased by 1.64 to 1.82 fold when transcription of *rne* was interfered. In contrast, *pks5_1* (*mmar_2344*), which is not regulated by B11, showed no significant difference in fold change when RNase E levels were reduced (1.53 vs 1.55). Interestingly, *udgL*, which had the highest affinity for B11 among the tested targets in our EMSA (Figure S6), displayed results consistent with our hypothesis that RNase E is involved in B11‐mediated mRNA decay. Specifically, its mRNA level remained repressed by B11 in the RNase E‐reduced strain (pB11 + pJR962‐*rne*), but was significantly higher than in the strain without the *rne*‐interference plasmid (pB11 + pJR962). This resulted in a reduced fold‐change (4.96 vs 2.95), suggesting that RNase E may be required for B11‐mediated regulation of *udgL*. However, for the other three tested genes (*mmar_2341*, *pks5*, and *papA3*), we did not observe significant differences in their mRNA levels between the two strains with B11 expression (pB11 + pJR962 vs pB11 + pJR962‐*rne*), regardless of the presence or absence of *rne* interference. The reduced fold changes for these three genes likely stem from a decrease in their mRNA levels in the *rne*‐interfered strain without B11 expression (pCtr + pJR962‐*rne*), suggesting a more complex and still poorly understood mechanism for B11‐mediated mRNA degradation.

## DISCUSSION

The extractable glycolipids of mycobacteria resemble the LPS of Gram‐negative bacteria, both of which are characterized by genus‐conserved fatty acids (mycolic acid or lipid A) in the inner leaflets and species‐specific lipids or glycans in the outer layer. Considering that LOS and LPS play key roles in adapting to varied environments and interacting with the host immune system, it is not surprising that their biosynthesis and assembly are controlled by multiple layers of regulation. In *E. coli* and many other Gram‐negative bacteria, the regulation of LPS production is controlled both at the transcriptional level by transcriptional regulators and at the posttranscriptional level by sRNAs^41^. For example, in *E. coli*, sRNA MgrR can fine‐tune LPS structure by repressing the LPS‐modification enzyme EptB^42^. Another sRNA, MicF, regulates lipid A modification by promoting the degradation of *lpxR* mRNA, which encodes lipid A deacylase.^43^ In *V. cholerae*, sRNA VadR can regulate peptidoglycan integrity and cell shape by repressing expression of periplasmic protein CrvA^44^. Although several conserved sRNAs have been identified in mycobacteria, only a few have been studied extensively for their biological roles and regulatory mechanisms, and none have been shown to be involved in cell wall synthesis. In this study, we demonstrated sRNA‐based regulation of cell wall synthesis in mycobacteria by performing a systematic identification of sRNA B11 targets. Through the use of several different approaches, including RNA sequencing and mass spectrometry screening, qPCR confirmation, MS2 affinity purification, and EMSA, we were able to identify genes involved in LOS synthesis that are regulated by B11. Although we did not analyze cell wall lipids to provide direct evidence of altered LOS production in the B11‐deleted strain, its altered colony morphology is consistent with previously reported phenotypes in strains with deletions in LOS synthesis genes *udgL* ^45^, ^46^, *pks5* ^5^, *fad25* (*mmar_2341*), and *papA3* ^33^. For example, a previous study found that inactivation of *udgL* (*mmar_2309*) and *mmar_2332* altered the colony morphology, resulting in wide translucent colony halos^46^, and deletion of *mmar_2331* or *wbbL2* also caused an extended translucent border compared to the wild‐type strain^47^. This observation is consistent with our findings that the B11‐overexpressing strain, which mimics a LOS‐deficient phenotype, also showed an extended translucent border compared to the B11 knock‐out strain, where LOS synthesis is upregulated, thereby supporting that B11 acts as a negative posttranscriptional regulator of LOS synthesis in *M. marinum*.

Because B11 is the most widely studied mycobacterial sRNA, it is possible to compare its function across different mycobacterial species, including *M. tuberculosis* ^19^, *M. smegmatis* ^26^, *M. abscessus* ^27^, and *M. kansasii* ^48^. However, the phenotype of B11‐deficient strains has only been described in *M. abscessus*, *M. kansasii,* and, here, in *M. marinum*. Inactivation of B11 by transposon insertion in *M. kansasii* resulted in colonies with a reduced diameter, abnormal surface topology, and a smooth and glistening appearance that contrasted with the rough and dried phenotype of wild‐type colonies. Whether this mutant phenotype in *M. kansasii* is also caused by the loss of B11 repression of LOS biosynthesis is currently unknown, as the study did not identify B11 target genes^48^. However, the genomic LOS biosynthesis locus in *M. kansasii*, particularly the *pks5* region, resembles its orthologue in *M. marinum* ^5^. Furthermore, we identified a G‐stretch close to the translation start site of the *M. kansasii pks5* gene *(mkan_27485*, an orthologue of *mmar_2340)*, suggesting that B11 regulation in *M. kansasii* could be similar to the B11 regulation we describe in *M. marinum*. In *M. abscessus*, which does not produce LOS, disruption of B11 resulted in smooth‐to‐rough colony variation by reducing the biosynthesis of GPLs through a mechanism, perhaps indirect, that appears to be independent of base‐pairing^27^. In addition, overexpression of B11 in the wild‐type background of *M. tuberculosis* and *M. smegmatis* resulted in elongated bacilli, a phenotype that may be associated with the composition of the cell wall^19^, ^26^. Thus, although B11 appears to be involved with the regulation of cell wall synthesis in several mycobacteria species, the targeted genes and the regulatory mechanisms vary.

One surprising finding is that the direct B11 targets are distinct in the three species studied with target screening: *M. smegmatis* ^26^, *M. abscessus* ^27^, and *M. marinum*. The direct B11 targets in *M. smegmatis* are involved with DNA replication and protein secretion, and in *M. abscessus* with ESAT‐6 secretion system (ESX)‐related genes, but we found no evidence that B11 is involved with the regulation of these processes in *M. marinum*. Curiously, while it appears that the sequences of both the promoter and regulatory base‐pairing seed regions of B11 are evolutionarily conserved in different *Mycobacterium* species, and even in other *Mycobacteriales* families such as *Nocardiaceae* and *Gordoniaceae*, the biologic processes regulated are distinct in the different species. This raises the question: if B11 exhibits distinct behavior across various *Mycobacterium* species, how does it maintain conservation throughout evolution? Studies on the conserved sRNA RyhB in *E. coli* and *Vibrio* suggest a model wherein broadly distributed sRNAs like RyhB play a core role in iron homeostasis that is conserved across species^49^, ^50^. However, the *V. cholerae* sRNA has acquired additional physiological roles in both aerobic and anaerobic respiration^51^, ^52^. Drawing from this, we postulated that an unfolded core function of B11 may underpin its conservation among different mycobacteria. This function appears to deviate from the canonical mode of action through RNA‐mRNA base pairing, potentially involving direct interaction with proteins. Further investigations are warranted to reveal this core function and ascertain whether B11 acts as a dual‐function sRNA.

Our data showed that the seed region for regulatory base pairing by B11 is a cytosine‐rich loop near its 3′ end, which appears to be conserved in the B11 orthologues in *M. smegmatis* ^26^ and *M. abscessus* ^27^. The use of a cytosine‐rich loop in the terminator as the pairing region is a characteristic feature of sRNAs in Gram‐positive bacteria, but it is relatively rare in Gram‐negative bacteria, with only two reports in *Helicobacter* and *Salmonella* ^53^, ^54^. Because mycobacteria have high GC‐content with numerous guanine‐rich motifs throughout the entire genome, computational methods using the cytosine‐rich regions to predict the targets were not accurate. For instance, RNApredator predicted 5423 binding sites for B11 (Table S3), and the locations of these sites covered almost all the annotated genes of *M. marinum*. Approximately 145 of these sites are predicted to have high stability (ΔG < −15). However, none of the genes associated with these 145 sites were identified in either our RNA sequencing or mass spectrometry data. In contrast, the primary target genes we identified, such as *udgL* and *mmar_4171*, were predicted to have relatively lower B11 binding (ΔG −12.01 and −10.55) and were not listed among the top candidates by RNApredator. Similarly, bioinformatic tools did not predict favorable binding energies for any of the experimentally confirmed targets of mycobacterial sRNA MrsI^25^. The mode of sRNA‐mRNA interaction in high GC‐content mycobacteria may be different from that in other prokaryotes, perhaps requiring less favorable binding energy and independent of RNA chaperones such as Hfq and ProQ, which have not been found in mycobacterial genomes. Therefore, attempts to identify targets of mycobacterial sRNAs by bioinformatic scanning the genome for potential base‐pairing sequences may be less fruitful than methods, such as those employed here, that can experimentally demonstrate sRNA‐mRNA interactions. Methods similar to RIL‐seq that are used in many bacteria^55^, ^56^, ^57^, yet independent of capturing RNA‐binding proteins, such as RIC‐seq utilized in eukaryotic cells^58^, are imperative for unraveling the rules governing sRNA‐mRNA interactions in mycobacteria.

We found that RNase E is likely involved in B11‐mediated degradation of its targets, at least for *udgL*. However, our data only support a partial involvement of RNase E in B11‐mediated mRNA degradation, as the fold changes were reduced by less than twofold, despite the fact that we observed no change for the negative control *pks5_1*. This may be due to limitations in our system, although we observed a ~10‐fold reduction of *rne* mRNA levels after interfering, we cannot exclude the possibility that changes at the protein level were minor. Additionally, since qRT‐PCR only measures a subset of mRNA abundance, and recent work has shown that incomplete transcripts dominate the *Mycobacterium* transcriptome^59^, a more accurate evaluation, such as RNA‐seq, would be more suitable for assessing the mRNA abundance of the entire transcript. Furthermore, the differential behavior of B11 target mRNAs upon *rne* interference suggests that sRNA‐mediated mRNA decay may not be exclusively mediated by RNase E but also by other complex mechanisms that need to be further explored in future work.

## MATERIALS AND METHODS

### Bacterial strains

A list of bacterial strains used in this study is shown in Table S4. *M. marinum* M strain (ATCC BAA‐535) was used as the wild‐type strain. Strains were grown at 30°C in 7H9 medium (~105 rpm) or 7H10 agar supplemented with 10% OADC and hygromycin B (20 μg/ml), kanamycin (25 μg/ml), or gentamycin (20 μg/ml) when necessary.

### DNA/RNA oligonucleotides and plasmids

Plasmids used in this study are shown in Table S5, and the sequences of the oligonucleotides used are listed in Table S6. Competent *E. coli* DH5α were used for cloning. Plasmids were isolated using the TIANprep Rapid Mini Plasmid Kit. For the generation of long DNA fragment (greater than 120 nt), such as the *pks5* 5′ UTR with compensatory mutations in the B11‐targeted region, DNA synthesis was carried out by Shanghai Saiheng Biotech Co. Ltd.

### Sequence alignments

Nucleotide blast search was performed with the following genomes: *M. marinum* M (NC_010612.1); *M. ulcerans* Agy99 (NC_008611.1); *M. kansasii* ATCC 12478 (NC_022663.1); *M. tuberculosis* H37Rv (NC_000962.3); *M. tuberculosis* H37Ra (NC_009525.1); *M. canettii* CIPT 140070007 isolate STB‐I (NZ_CAOO00000000.1); *M. bovis* BCG str. Tokyo (AP010918.1); *M. africanum* K85 (KK338483.1); *M. avium* subsp. *paratuberculosis* K‐10 (NC_002944.2); *M. intracellulare* ATCC 13950 (NC_016946.1); *M. indicus pranii* MTCC 9506 (CP002275.1); *M. smegmatis* str. MC2 155 (NC_008596.1); *Mycolicibacterium vanbaalenii* PYR‐1 (NC_008726.1); *Mycolicibacterium gilvum* Spyr1 (NC_014814.1); sequence alignments were generated online in website http://multalin.toulouse.inra.fr/multalin/multalin.html.

### Construction of the Δ*b11* strain

Deletion of B11 in *M. marinum* was achieved by a modified allelic exchange method using the temperature‐sensitive pPR27 plasmid^32^. This vector harbors a thermosensitive origin of replication and the *sacB* gene from *B. subtilis*, which is lethal to mycobacteria in the presence of sucrose. However, the temperature generally used for losing the plasmid, 39°C, is not suitable for *M. marinum* growth. To solve this, we inserted the *mWasabi* gene to create a pPR27‐*mWasabi* plasmid that expresses a brighter green fluorescent protein. *M. marinum* strains carrying this plasmid have a green color that distinguishes them from white strains that have lost the plasmid, which made it possible to perform the selection at 30°C. For allelic exchange, 1 kb downstream of b11 nucleotides 36–91 was amplified by PCR with oligo QGO‐037/038, and 1 kb upstream was amplified with oligo QGO‐039/040. The two fragments were fused on either side of a kanamycin resistance gene amplified from the pMV306 plasmid with oligos QGO‐041/042, to yield a ~3.2 kb fragment with the kanamycin resistance gene in the middle. The fused product was inserted into pPR27‐*mWasabi* and transformed into the wild‐type *M. marinum* M strain. After 10 days of growth, kanamycin and gentamicin double‐resistant, GFP‐positive colonies were selected from 7H10 plates and incubated in 7H9‐OADC media supplemented with kanamycin and gentamicin to OD_600_ ~ 0.5. The bacterial culture was collected and filtered through a 5 μM filter (Sartorius) to obtain a single‐bacterial cell suspension. The suspension was then serially diluted and plated on 7H10‐agar containing 10% sucrose to inhibit the growth of cells retaining the pPR27 plasmid. After 20 days, one colorless clone was found among ~5000 green colonies on 100 plates. After confirming the loss of fluorescence with the Typhoon FLA 9000 system (GE), the colorless clone was incubated in 7H9‐OADC supplemented with kanamycin to OD_600_ ~ 0.5 and then streaked on 7H10‐OADC plates to isolate a pure strain whose B11 deletion was confirmed by PCR and northern blot.

### RNA isolation and Northern blot analysis

Bacterial RNA was extracted using the TRIzol method. Briefly, 4 OD_600_ of bacterial cultures were harvested by centrifugation, and cell pellets were immediately resuspended in 1.2 ml of Trizol reagent (Invitrogen) and 0.25 ml of 0.1 mm Zirconium/Silica beads (Biospec). Bacterial cells were lysed using an OMNI Bead Ruptor 4 homogenizer (6000 rpm, 3 × 20 s, with 5‐min intervals on ice). 1 ml of the lysate was mixed with 400 μl of chloroform in a 2 ml Phase Lock Gel tube (TIANGEN Biotech), and centrifuged at 13,000 rpm for 20 min. The aqueous phase (500 μl) was collected and combined with 450 μl of isopropanol for RNA precipitation, which was allowed to occur for 30 min. The RNA pellet was collected by centrifugation, washed with 75% ethanol, and then dissolved in DEPC‐treated distilled water.

For northern blot, 5–10 μg of RNA was denatured at 95°C in gel loading buffer II and separated on 6%–8% PAGE‐urea gels in TBE buffer. RNA was transferred to Hybond‐N+ membranes by electroblotting for 1 h at 50 V. The membranes were prehybridized in DIG Easy Hyb solution (Roche) for 30 min and then hybridized overnight at 50°C with a Digoxin‐labeled probe synthesized in Saiheng Biotech. After hybridization, the membranes were washed sequentially for 20 min each in 5× SSC/0.1% SDS, 1× SSC/0.1% SDS, and 0.5× SSC/0.1% SDS. The membrane was incubated in maleic acid buffer (0.1 M maleic acid, 0.15 M NaCl, 0.3% Tween‐20, pH 7.5) for 10 min, followed by blocking in blocking solution for 60 min at 37°C. The membrane was then incubated with 75 mU/ml Anti‐Digoxigenin‐AP (Roche) in blocking solution for 60–120 min. After washing with maleic acid buffer, the membrane was equilibrated in detection buffer, and signals were visualized using CDP‐star (Roche) on a ChemiDoc™ MP station.

### RNA sequencing

RNA sequencing was performed by BGI Group, China. Two biological replicates from each group were analyzed. A strand‐specific cDNA library was prepared, and sequencing was carried out on a BGISeq‐500 platform. After adapter removal, the quality of the data was assessed using the SOAP package^60^. High‐quality reads were aligned to the *M. marinum* M genome (NC_010612.1) using HISAT software^61^. Gene expression levels were calculated using Bowtie2 and RSEM packages. The RNA levels were quantified using fragments per kilobase of transcript per million mapped reads (FPKM)^62^, ^63^.

### Mass spectrometry

The mass spectrometry proteomics analysis was performed by Shanghai iProteome Biotechnology Co. Ltd. Three biological replicates for each group were analyzed. Briefly, ~50 OD_600_ bacteria cells cultivated in 7H9‐OADC medium were collected and washed three times with phosphate buffer saline (PBS) buffer. Samples were lysed in lysis buffer (8 M urea, 100 mM Tris, pH 8.0) containing protease inhibitors and sonicated for lysis. The lysates were centrifuged to collect supernatants. 1 mg of protein was reduced with dithiothreitol at 56°C and alkylated with iodoacetamide at room temperature, and digested with trypsin. Tryptic peptides were vacuum‐dried and analyzed on an HFX Hybrid Quadrupole‐Orbitrap Mass Spectrometer (Thermo Fisher Scientific, Rockford, IL, USA) coupled with a high‐performance liquid chromatography system (EASY nLC 1200, Thermo Fisher). The original data from the mass spectrometry analysis were .d files, and MaxQuant was used for qualitative and quantitative analysis. The fraction of total (FOT), a relative quantification value, was defined as a protein's intensity‐based absolute quantification^64^.

### Quantitative PCR (qPCR)

qPCR was conducted using the PrimeScript™ RT Reagent Kit (Takara Bio). Total RNA (1 μg) was treated with DNase and reverse‐transcribed into cDNA following the manufacturer's instructions. The PCR was performed with TB Green Premix Ex Taq II (Takara Bio) using the Biorad CFX96 Touch Real‐Time PCR Detection System. Data were normalized using the relative quantification method, with the *rfaH* gene serving as the reference.

### B11 transcript synthesis and labeling

B11 transcripts were synthesized in vitro using 200 ng of a DNA fragment amplified from *M. marinum* genomic DNA with primers QGO‐782/784, serving as the template in a T7 transcription reaction with the T7 Transcription Kit (Thermo Fisher Scientific). The size and integrity of the RNA were confirmed through PAGE gel electrophoresis. The RNA was excised from the gel, eluted in 0.1 M sodium acetate, 0.1% SDS, and 10 mM EDTA at 4°C overnight, and then subjected to phenol:chloroform:isoamyl extraction and ethanol precipitation. For radio‐labeling, 50 pmol RNA was dephosphorylated using 10 units of calf intestine alkaline phosphatase in a 50 μl reaction at 37°C. After phenol:chloroform:isoamyl extraction, the dephosphorylated RNA was 5′‐labeled with 3 μl of 32P‐γ‐ATP using T4 polynucleotide kinase. Unincorporated nucleotides were removed by centrifugation using Microspin G‐50 columns (GE Healthcare).

### RNA structure probing

Structure probing was carried out on in vitro transcribed 5′‐radio‐labeled B11 as previously described^17^. 0.2 pmol of radio‐labeled RNA was denatured at 95°C for 2 min and then cooled on ice. Subsequently, yeast tRNA and 10× structure buffer (0.1 M Tris–HCl, pH 7, 1 M KCl, 0.1 M MgCl_2_) were added, followed by incubation at 37°C for 30 min. The samples were then treated with 5 mM lead (II) acetate for 1.5 min. For RNase T1 sequencing ladders, RNA was digested with 0.1 U RNase T1 for 5 min at 37°C. Alkaline sequencing ladders were prepared by incubating RNA at 95°C for 5 min in alkaline hydrolysis buffer. Reactions were stopped by adding Gel Loading Buffer II. The samples were denatured at 95°C and resolved on 8% denaturing sequencing gels containing 1× TBE at a constant power of 40 W. Gels were dried and analyzed using a Typhoon FLA 9500 phosphoimager.

### MS2 affinity purification

MS2 affinity purification was performed as described^37^. MS2‐B11 expressed from the *hsp60* promoter showed expression and regulatory ability similar to B11 expressed from its native promoter; the Δ*b11* + pP*_b11_*‐B11 strain was used as the control for the affinity purification. Briefly, 50 OD_600_ bacterial cultures were washed and resuspended in 10 ml ice‐cold buffer A (20 mM Tris–HCl at pH 8.0, 150 mM KCl, 1 mM MgCl2, 1 mM DTT, and 1 mM PMSF) and frozen immediately in liquid nitrogen. These samples were mixed with 0.1 mm Zirconia/Silica beads (Biospec) and disrupted with a Precellys 24 homogenizer (6800 rpm, 5 × 30 s, separated by 10 min intervals in liquid nitrogen). The lysates were cleared by centrifugation at 15,000 rpm, 4°C for 30 min, and supernatants were collected. Before affinity purification, 75 μl amylose resin (#E8021S, New England Biolabs) was added to a Bio‐Spin disposable chromatography column (Bio‐rad) and equilibrated three times with 1 ml Buffer A. 100 pmol purified MS2‐MBP coat protein was then diluted in 1 ml Buffer A and applied onto the column. After 5 min incubation and two column washes with Buffer A, 10 ml of bacterial lysate was loaded onto the column. The column was washed five times with 1 ml Buffer A and then eluted with 1 ml Buffer A supplemented with 15 mM maltose. Eluted RNA was isolated by phenol:chloroform:isoamyl extraction and precipitation as described for RNA labeling. 50 ng RNA, quantified with a 5300 Fragment Analyzer System (Agilent), was then used for qRT‐PCR reactions.

### Electrophoretic mobility shift assays (EMSA)

For the EMSA, 0.5 pM of in‐vitro transcribed B11 RNA was denatured at 95°C for 2 min, followed by rapid cooling on ice for 5 min. The RNA was then mixed with varying concentrations of in‐vitro transcribed targeted mRNA 5′ UTR in a final reaction volume of 10 μl. The buffer conditions were 10 mM Tris–HCl (pH 7), 100 mM KCl, and 10 mM MgCl₂. The reactions were incubated at 37°C for 30 min and then stopped by adding 5x RNA native loading buffer. Samples were resolved on 6% native polyacrylamide gels at 4°C in 0.5× TBE buffer under a constant current of 40 mA for 4 h. After electrophoresis, RNA was visualized by staining the gels with SYBR Green II (#S7564, Thermo Scientific) and imaged using a ChemiDoc™ XRS+ system. Quantification was performed using ImageLab™ Software (Bio‐Rad).

### Bioluminescence assay

Bacterial cultures at OD_600_ ~ 1.0 were serially diluted and plated on 7H10 ‐OADC agar. For the bioluminescence assay, 0.1 ml of culture was added into the wells of white 96‐well plates (#3917, Corning). 1% decanal (#D7384, Sigma‐aldrich) in ethanol was diluted 1:25 with 7H9 medium to a final concentration of 0.04% to make the reaction buffer. Subsequently, 0.1 ml reaction buffer was added to the wells with bacterial cultures, and bioluminescence was detected by using a FlexStation 3 Multi‐Mode Microplate Reader (Molecular Devices).

### Statistical analyses

Statistical parameters for each experiment are detailed in the respective figure legends. Statistical analysis was conducted using GraphPad Prism 9 (GraphPad Software). Experiments were not blinded or randomized, and no prior estimation of statistical power was conducted. Additionally, no data were excluded from the analysis.

## AUTHOR CONTRIBUTIONS

**Chuan Wang**: Data curation; funding acquisition; methodology; supervision; writing—original draft; writing—review and editing. **Cheng Bei**: Methodology. **Yufeng Fan**: Data curation; methodology. **Qingyun Liu**: Data curation; methodology. **Yue Ding**: Data curation; methodology. **Howard E. Takiff**: Writing—review and editing. **Qian Gao**: Supervision; writing—review and editing.

## ETHICS STATEMENT

No animal experiments nor human samples were involved in this study.

## CONFLICT OF INTEREST

The authors declare that they have no conflict of interest.

## Supporting information

Supplemental Tables.

Supplementary figures.

## Data Availability

The data supporting the findings of this study are available from the corresponding authors upon request. The RNA sequencing data have been deposited in the GEO database under No. GSE264453. Mass spectrometry data have been deposited in the iProX database under PXD051635.
