# Supplementary material for: Conserved mycobacterial sRNA B11 regulates lipooligosaccharide synthesis at posttranscriptional level in Mycobacterium marinum
Source: mLife. 2025 Aug 25;4(4):447–60. doi: 10.1002/mlf2.70025 (PMC12395582; doi:10.1002/mlf2.70025)
Supplement: Supplementary file 2 — Supplementary figures. [file MLF2-4-447-s002.pdf]

Figure S1

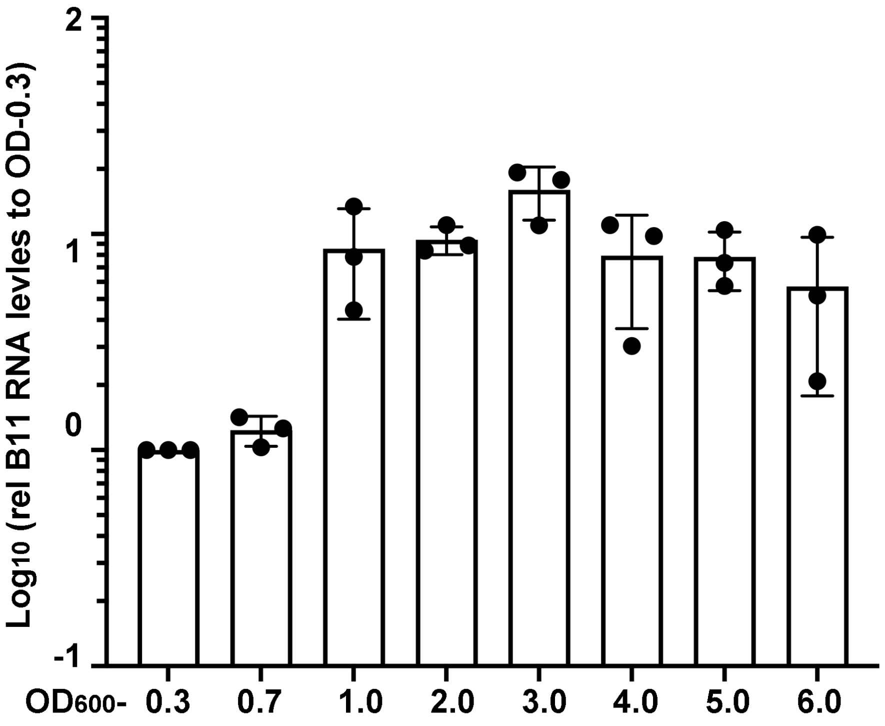

Figure S1. Expression levels of B11 in *M. marinum* wild-type strain during standard 7H9-OADC growth, quantified by qRT-PCR. *sigA* RNA was used as reference gene. Expression was normalized to the B11 RNA level at OD_{600} ~0.3 (n = 3).

Figure S2

(A)

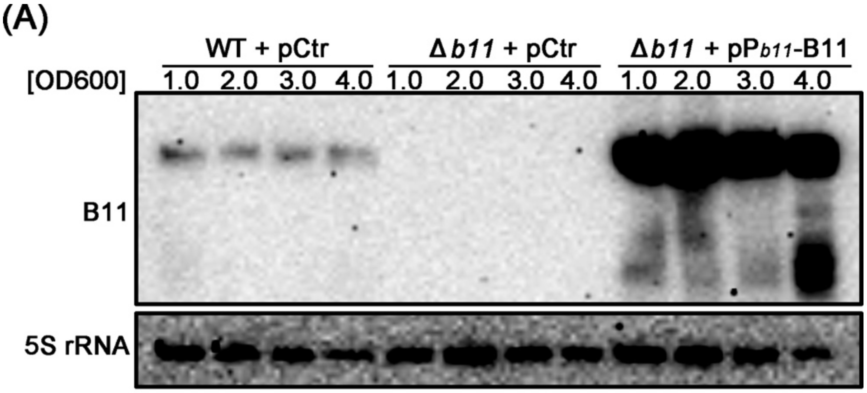

(B)

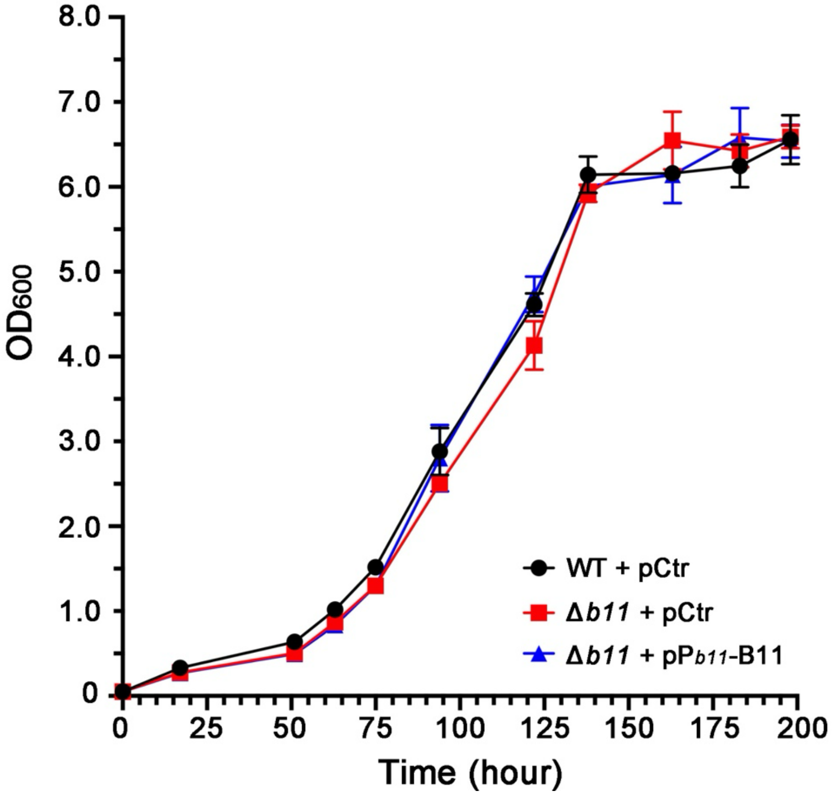

Figure S2. (A) Expression levels of B11 in *M. marinum* wild-type (WT + pCtr), B11-deleted (Δ*b11* + pCtr) and complemented (Δ*b11* + pP$_{b11}$-B11) strains. Northern blot analysis of total RNA isolated from different strains grown to the indicated OD$_{600}$. 5S RNA was used as loading controls. (B) Growth curve of indicated *M.marinum* strains in 7H9-OADC. Error bars indicate standard deviations (n = 3).

Figure S3

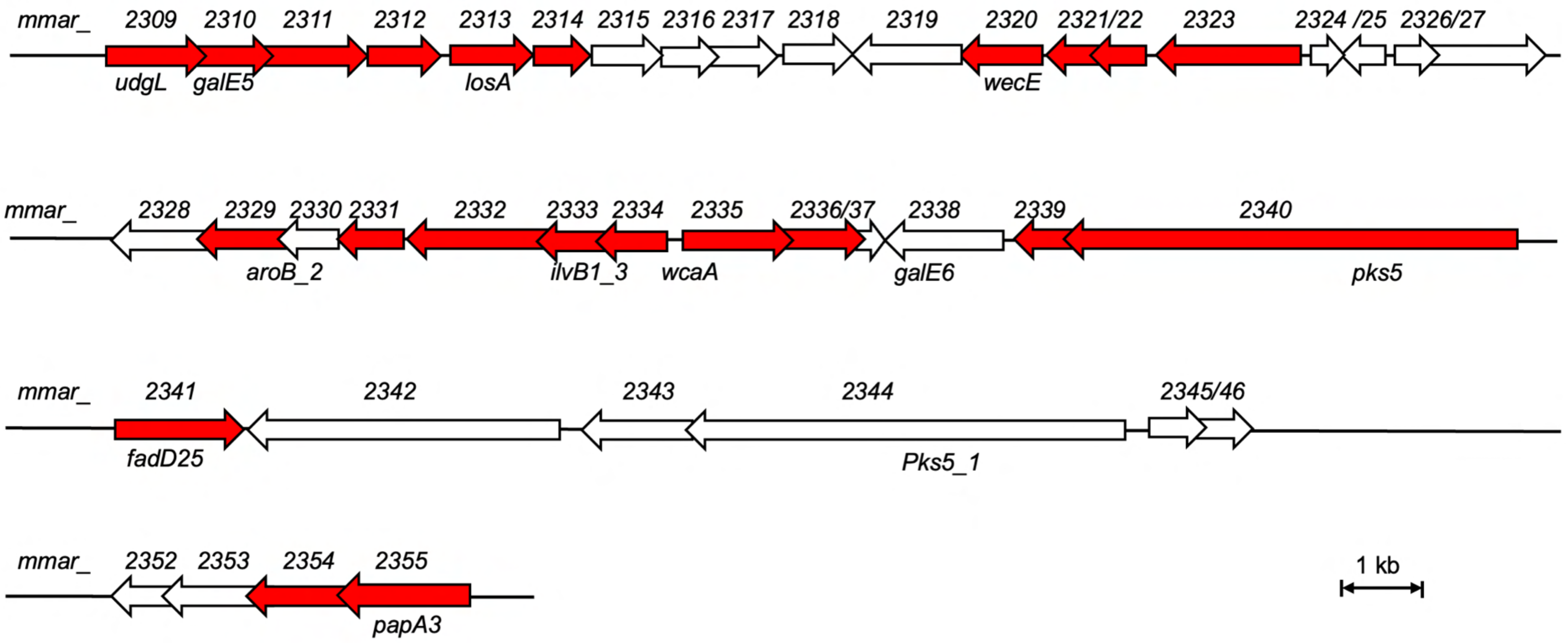

Figure S3. Genetic organization of the LOS biosynthetic locus (*mmar_2309-mmar_2346* and *mmar_2352-mmar_2355*) of *M. marinum.* Genes exhibiting significant changes ($p<0.05$) between Δ*b11* + pCtr and Δ*b11* + pP$_{b11}$-B11 strains, as identified by both RNA-seq and mass spectrometry, are highlighted in red.

Figure S4

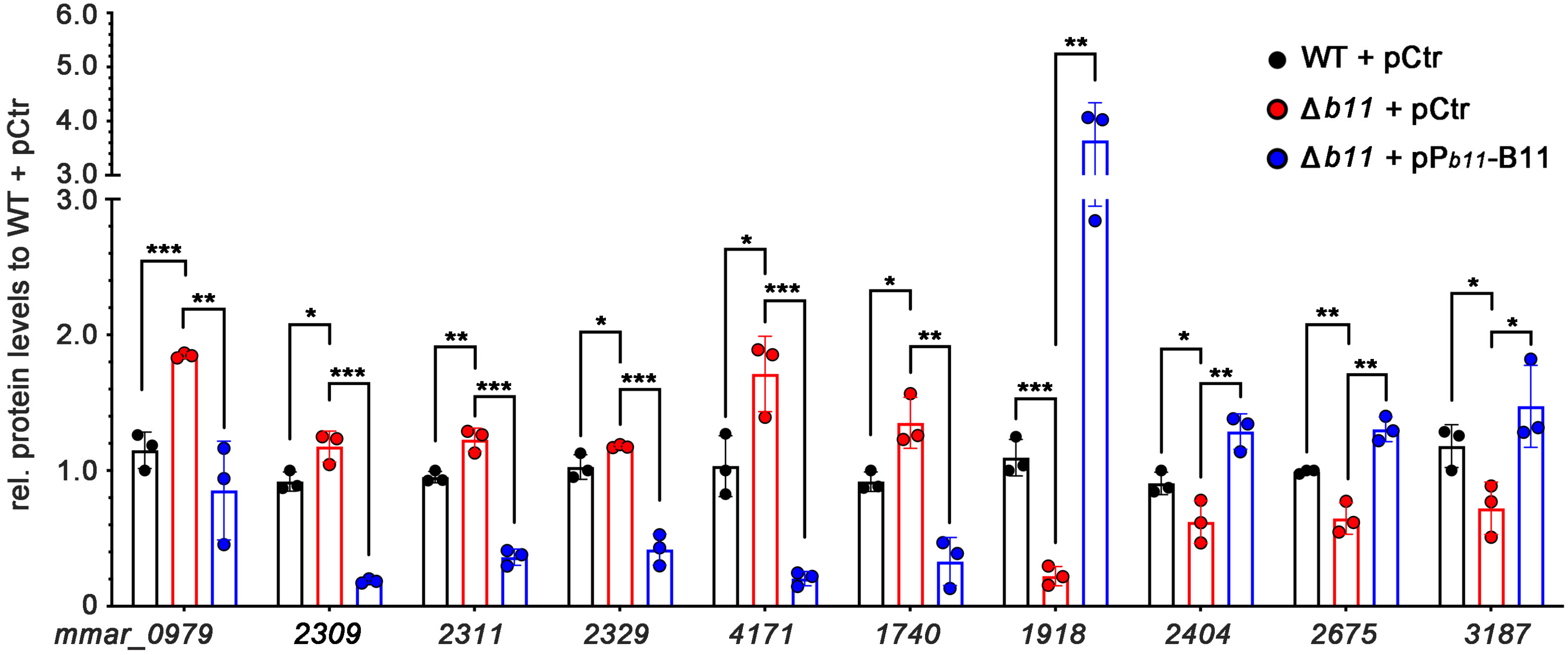

Figure S4. The top 10 regulated and abundant proteins identified by mass spectrometry. Protein expression levels in the specified strains were assessed via mass spectrometry and normalized to the expression of a sample from the WT + pCtr group. Significance levels: * p<0.05, ** p<0.01, *** p<0.001; ns indicates no significant difference, determined by a two-tailed t test (n=3).

Figure S5

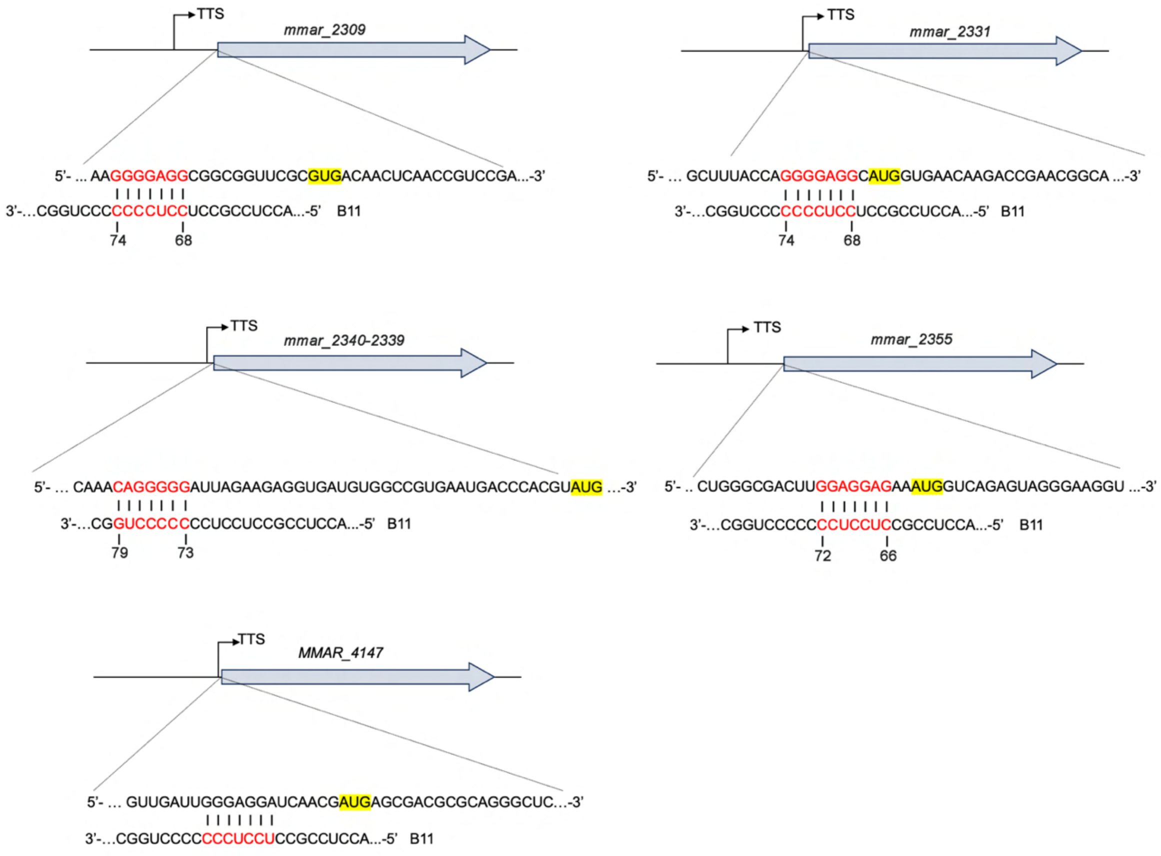

Figure S5. IntaRNA-predicted binding sites between B11 and targeted genes. Start codons are highlighted, and base-pairing regions are depicted in red. Transcriptional start sites (TTS) were determined using published differential RNA-seq data (mBio. 2014; 5(4): e01169-14). The base positions of the B11 critical base-pairing region are annotated numerical.

Figure S6

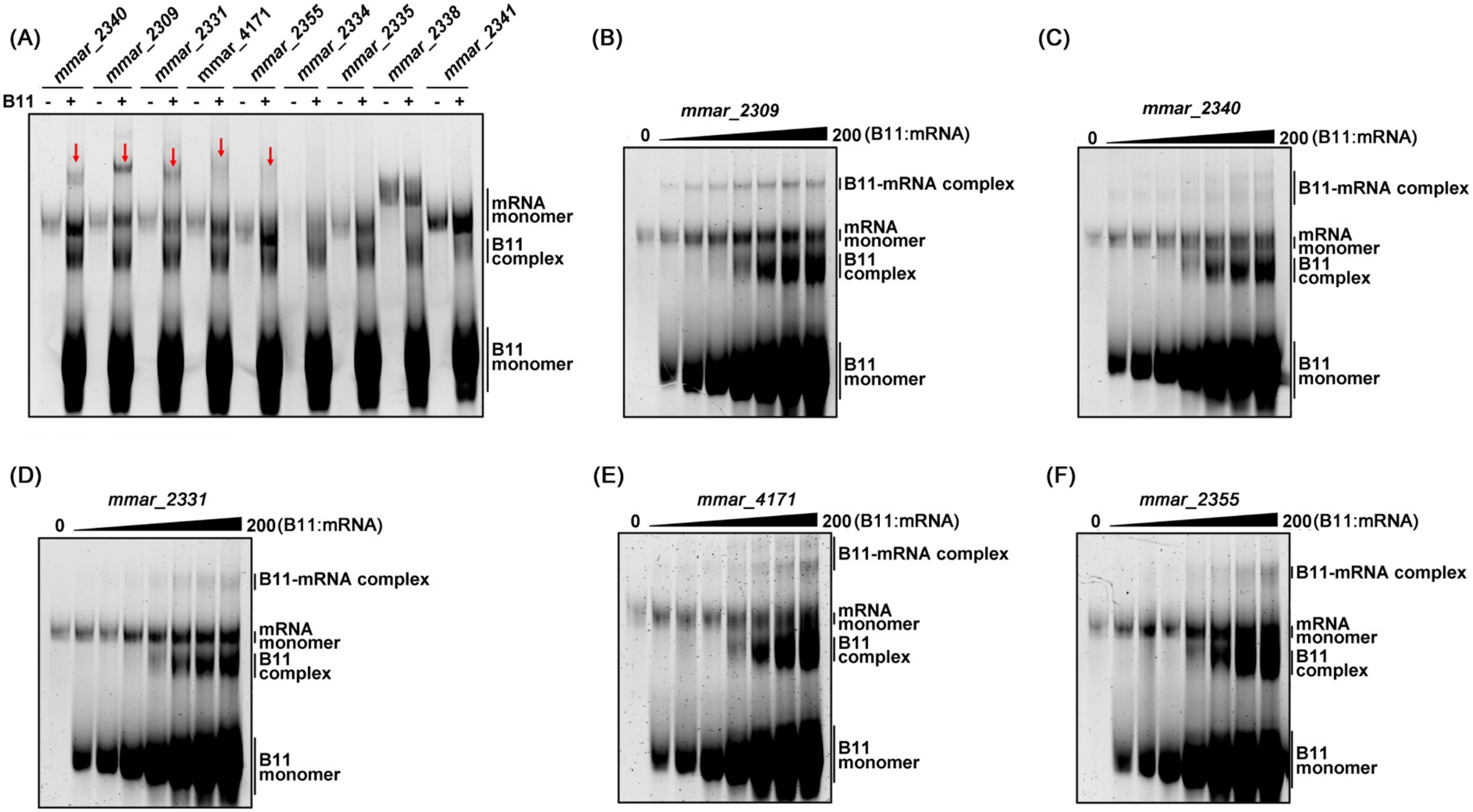

Figure S6: Electrophoretic Mobility Shift Assay (EMSA) of B11 Interacting with mRNAs. (A) SYBR Green II-stained PAGE gel showing the 5' end of nine selected mRNAs (0.5 pmol/reaction) alone or incubated with B11 at a molar ratio of 100:1 (B11: mRNA), the red arrows correspond to the shifted B11-mRNA complex. (B-F) SYBR Green II-stained PAGE gel showing the 5' end of 5 B11-bound mRNAs (0.5 pmol/reaction) incubated with B11 at increasing molar ratios (0/5/10/20/50/100/150/200:1 from left to right, B11: mRNA) as indicated above the gel. Representative of three independent experiments.

Figure S7

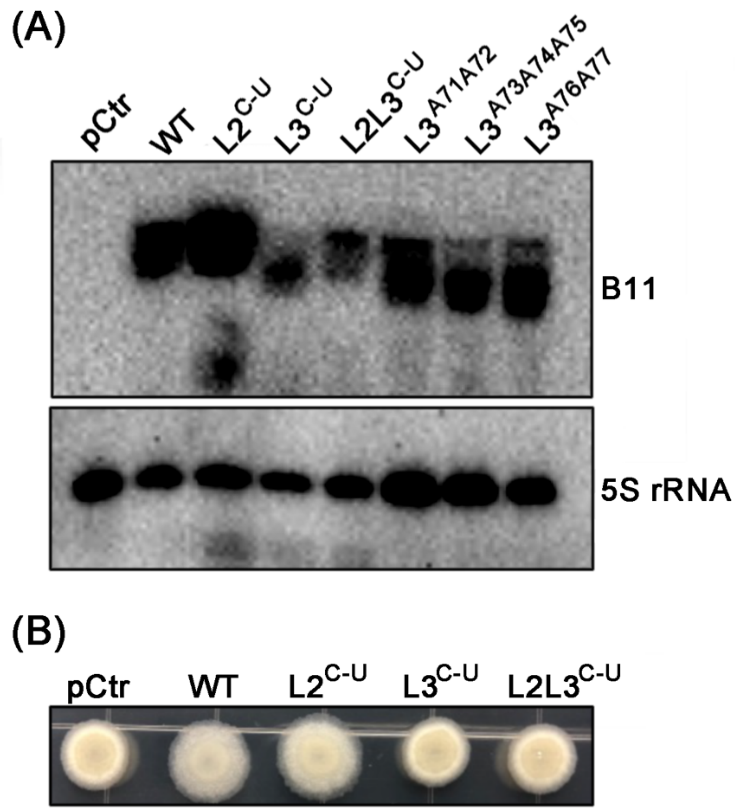

Figure S7. (A) Northern blot analysis of total RNA isolated from B11-deleted strain carrying different plasmids. 5S RNA was used as loading controls. (B) Morphology of indicated B11-deleted strain carrying indicated plasmids in 7H10 agar. Representative of three independent experiments.

Figure S8

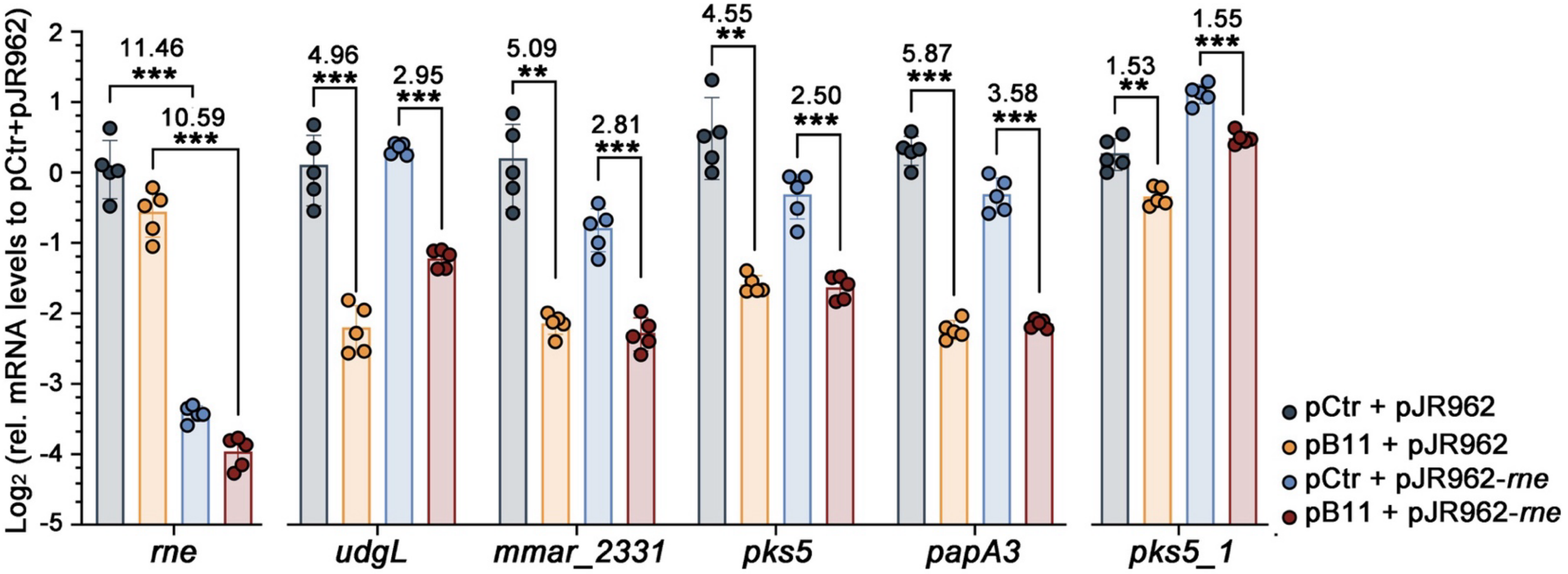

Figure S8. B11-mediated mRNA repression is likely RNase E-dependent. Expression of *rne* and selected B11-target genes *udgL*(*mmar_2309*), *mmar_2331*, *pks5*(*mmar_2340*), *papA3* (*mmar_2355*) in *M. marinum* strains containing either a control pSMT3 empty plasmid pCtr or a B11 overexpression plasmid pB11 (pP_{hsp60}-B11), and either the control plasmid pJR962 or an *rne*-interference plasmid pJR962-*rne*. The non-B11 target *pks5_1* (*mmar_2344*) was also checked as a negative control. Expression levels were normalized to one sample from the pCtr + pJR962 group. 16S rRNA instead of *sigA* was used as the reference gene for data analysis, as *sigA* expression was also affected by *rne* interference. Bacterial cultures were grown to OD_{600}~1.0, treated with ATc, and cultured for 24 hours prior to RNA sample collection. Fold changes in B11-mediated repression (pB11 / pCtr) are indicated above the bars. Error bars represent standard deviations (n = 5). **, p<0.01; ***, p<0.001, determined by a two-tailed t test.
